# Bridging the gap in pediatric nutrition: a narrative review of nutritional support strategies and their impact on child health inequalities

**DOI:** 10.3389/fpubh.2026.1850304

**Published:** 2026-09-15

**Authors:** Daqin Wu, Dong Huang

**Affiliations:** Pediatric Critical Care Medicine, Guizhou Provincial People's Hospital/Shanghai Children's Medical Center GuiZhou Hospital, Shanghai Jiao Tong University School of Medicine, Guizhou, Guiyang, China

**Keywords:** cognitive development, health, nutrition, pediatric nutrition, public health

## Abstract

Childhood malnutrition is an urgent public health concern that negatively influences vulnerable populations and strengthens inequities in health. Child malnutrition occurs both as a result of undernutrition and overnutrition, both of which disrupt child physical growth and mental development and, in turn, lead to long-term health consequences that may extend into adulthood, as well as perpetuate cycles of malnutrition intergenerationally. The purpose of this narrative review is to evaluate nutritional support strategies through locally relevant policies, community engagement and support, health care systems, and household-level supports to assess their effectiveness in reducing disparities in child health outcomes. Evidence has demonstrated that policy, community, and health-care-integrated strategies have been most effective in reaching disadvantaged children. Examples of effective policy strategies include food fortification, subsidies, and cash transfer initiatives, which improve the availability and access to adequate and appropriate foods for disadvantaged children. Community and household-level program strategies help improve the diversity of children’s diets, adherence to eating programs, and caregiver practices. The quality of care improves when nutrition assessment and counseling are integrated into primary health care, thereby enhancing the early identification and management of malnutrition. Evidence indicates that culturally relevant, equity-focused, and multi-level interventions produce the greatest improvements in child nutrition and health equity. Therefore, context-specific and sustainable strategies are vital for closing the childhood nutrition gap and ultimately ensuring those children’s growth, cognitive development, and long-term health outcomes are optimal throughout the world.

## Background

Nutritional deficiencies in early childhood affect a child’s physical growth, how their brain develops, and ultimately whether they will be healthy as an adult (1). Improvements in healthcare, food security, and child survival have contributed to overall progress; however, persistent socioeconomic inequalities, limited access to nutritious foods and healthcare, and inadequate nutrition services continue to contribute to a substantial burden of malnutrition in resource-limited settings. Nutritional deficits have been attributed to both morbidity and mortality in children. Stunting, wasting, and nutritional deficiencies affect many of the 149 million children under 5 years old (2). Malnutrition is one of the leading causes of immunologic defects and increased incidence of infection in children. Neurological development can also have lifelong effects on human capital development. Children who live in poverty and in rural areas have a much higher incidence of malnutrition and demonstrate that nutrition in children has numerous structural and social determinants associated with it (3). Based on this evidence, improving child health and nutritional status requires a multifaceted approach that addresses both biological nutritional needs and the broader social determinants of health (4).

Research has shown that nutrition is inequitable due to a combination of socioeconomic and environmental factors that affect a child’s ability to obtain an adequate diet and access healthcare (5). Children’s nutrition is affected by factors such as a household’s wealth, maternal education level, sanitation, location, and gender-based inequalities. Young children who are from poor households are much more likely to suffer from stunting and being underweight than children from wealthier households, as shown by demographic and health surveys conducted in many different countries (6). Basic structural factors like food insecurity, lack of a functional healthcare delivery system, poor maternal education, and limited availability of maternal health services in some countries, and differences in public health resources across regions further amplify these outcomes. For example, in Pakistan and India, there are rural – urban differences in nutrition, and many women do not receive adequate maternal health services, which compounds the inequities present in nutrition and health. Because these factors co-exist, children from disadvantaged families frequently experience several different types of risk factors at the same time. They are thereby at increased risk for developing inter-generational patterns of poor health and social inequity (7).

There are many different ways nutritional support strategies can affect child health through complex biological and social pathways. Properly implemented nutrition interventions help prevent malnutrition and improve children’s growth. Good nutrition also contributes to the development of the immune system, helps maintain the balance of intestinal microbiota, and decreases vulnerability to infectious disease, particularly diarrheal illnesses, which are highly related to childhood malnutrition. Additionally, to create long-lasting improvements in child health, nutrition-sensitive interventions such as improving access to safe water and sanitation, providing maternal education, and reducing poverty are also critical (8). Research shows that integrated strategies that combine both direct support for nutrition and addressing underlying social determinants can greatly reduce the burden of undernutrition and inequities in health based on social status (9). The interventions reach children by enhancing absorption of nutrients, providing greater strength to immunity with nutrient-rich foods, improving the quality of parenting behavior among mothers, and providing access to health care services. To develop effective public health interventions, it is also essential to have an adequate understanding of how nutrition and social determinants affect one another (10).

While much research has focused on pediatric nutrition, gaps still exist regarding how nutritional support strategies can help reduce health disparities among children. Many studies on nutrition have looked only at clinical outcomes or prevalence patterns, but have not adequately considered how different types of interventions impact equity among children of different socioeconomic statuses. There are also a few studies that synthesize the evidence on how nutrition-specific and nutrition-sensitive interventions fit together to help close the gap in child health outcomes. Recent studies show that difficulties related to social conditions, such as poverty, differences in education levels for mothers, and availability of health care in different geographic locations, continue to make it difficult for traditional nutrition programs to work effectively (11). In addition, much of the available literature does not provide a broader understanding of how the various biological mechanisms involved in nutrition, social determinants of health, and the policy-level aspects of children’s nutrition fit together in specific pediatric nutrition approaches. The purpose of this review is to address this gap in our understanding by assessing existing nutritional support interventions in pediatric patients, while also providing information on possible health disparities among children.

## Methodology

### Review design

In this study, a narrative review was carried out in order to consolidate the available evidence regarding nutritional support methods for children as well as to evaluate their impact on nutrition state of children, health results and health inequalities in children which is why the choice of narrative review design was made since literature was characterized by a variety of intervention approaches, groups of people, locations, and results that were impossible to combine in a quantitative manner. The focus of the review was on the methods of intervention that were implemented on the level of home, community, system of healthcare and at the level of regulation, it was especially important to provide information on differences in access and efficiency of the intervention methods in different economic or geographical groups.

### Review questions

The review was based on the following questions:

Which methods of nutritional support can be used in order to improve nutrition in pediatric age and health status of children?

What health outcomes can be achieved after the implementation of these interventions?

What are the socio-economic, environmental, cultural, and healthcare-related factors influencing access to the nutritional interventions?

### Literature search strategy

A comprehensive literature search was performed using PubMed/MEDLINE, Scopus, Web of Science, Embase, and Google Scholar. The time frame searched was between January 2000 and August 2026 to cover both traditional and novel forms of pediatric nutrition.

The search keywords were developed using various combinations of terms related to pediatric nutrition, malnutrition, nutritional assistance, health of children, inequalities in health, economic determinants, food supply problems, nutrition programs, supplements, breastfeeding, school meals, social safeguards, and community-based nutrition programs.

### Burden and determinants of nutritional inequalities

#### Global burden

Undernutrition and micronutrient deficiencies continue to impose significant health burdens on children globally, while the prevalence of overnutrition is increasing. The Global Burden of Disease studies show that children face high levels of suffering and death from nutritional deficiencies. In 2019, there were approximately 2,207.71 new cases of nutritional deficiencies per 100,000 people and an estimated 680.12 disability adjusted life years (DALYs) per 100,000 people due to the effects of malnutrition on child health. Children under 5 are the most severely affected population, suffering from high rates of both malnutrition and malnutrition-related diseases (12). The most significant contributors to the burden of malnutrition on child health are protein-calorie malnutrition, iron deficiency anemia, vitamin A deficiency, and iodine deficiency, all of which critically affect growth and development, cognition, immune function, and survival (13).

Childhood nutritional deficiencies around the world differ across regions and countries and illustrate the disparity in access and quality of healthy foods. The GBD analysis shows that South Asia and Sub-Saharan Africa have the highest rates of prevalence and disability adjusted life years (DALY) associated with childhood malnutrition (14). In contrast to South Asia and Sub-Saharan Africa, high-income regions (i.e., North America and Western Europe) have lower rates of classic micronutrient deficiencies; however, they are facing an ever-increasing incidence of overweight and obesity in children. In addition, countries with lower socio-demographic index (SDI) scores have significantly higher rates of protein-energy malnutrition and micronutrient deficiencies than do countries with higher SDI scores. For example, iron deficiency is the largest contributor to DALYs quantifying nutrition-related deficiencies around the world, and there is a low incidence of iron deficiency across low- and middle-income countries (15).

The global burden of overall micronutrient deficiency has been decreasing over the last two decades as a result of many expanded public health-related initiatives, including food fortification, supplementation programs, and better maternal and child health care services (9). However, the global burden of micronutrient deficiency remains high, and progress will continue to differ from nation to nation. The incidence of malnutrition in children under age five has declined substantially in high-income nations Still, in low-income countries, many continue to have limited or very small changes in terms of nutritional outcomes. Projected outcomes based on the GBD 2021 data indicate that although the average global burden of nutritional deficiencies is projected to decrease over the next 5 years, large differences among countries will be evident through the year 2035 unless enhanced policy initiatives and targeted nutrition programs are utilized. These new patterns underscore the immediate need for comprehensive nutrition strategies focused on equity to address the significant disparities in nutrition globally and improve child health (16) (Table 1, Figure 1).

**Table 1:** Key dimensions of pediatric nutrition importance.

Aspect	Description	Impact on child health	References
Physical growth	Adequate nutrients for height/weight	Prevents stunting	(68)
Brain development	Nutrients support neural growth	Improves cognition	(69)
Immunity	Vitamins and minerals strengthen immunity	Reduces infections	(70)
Energy balance	Macronutrients provide energy	Supports activity	(71)
Bone health	Calcium and Vitamin D role	Prevents rickets	(72)
Metabolism	Balanced diet regulates metabolism	Prevents obesity	(73)
Organ development	Supports organ maturation	Enhances function	(74)
Hormonal balance	Nutrition affects hormones	Growth regulation	(75)
Gut health	Fiber and microbiota interaction	Better digestion	(76)
Learning ability	Nutrition affects focus	Academic performance	(77)
Emotional health	Nutritional status affects mood	Behavioral stability	(78)
Lifelong health	Early nutrition shapes future health	Chronic disease risk	(79)
Muscle development	Protein supports muscle growth	Physical strength	(80)
Micronutrient sufficiency	Prevents deficiencies	Reduces anemia	(81)
Appetite regulation	Balanced diet stabilizes hunger	Prevents overeating	(82)
Sleep patterns	Nutrition influences sleep	Better recovery	(83)
Disease resistance	Antioxidants reduce illness risk	Health protection	(84)
Developmental milestones	Nutrition supports milestones	Timely development	(85)
Cognitive speed	Nutrients enhance brain function	Faster processing	(5)
Quality of life	Overall well-being improvement	Healthy childhood	(86)

**Figure 1:**
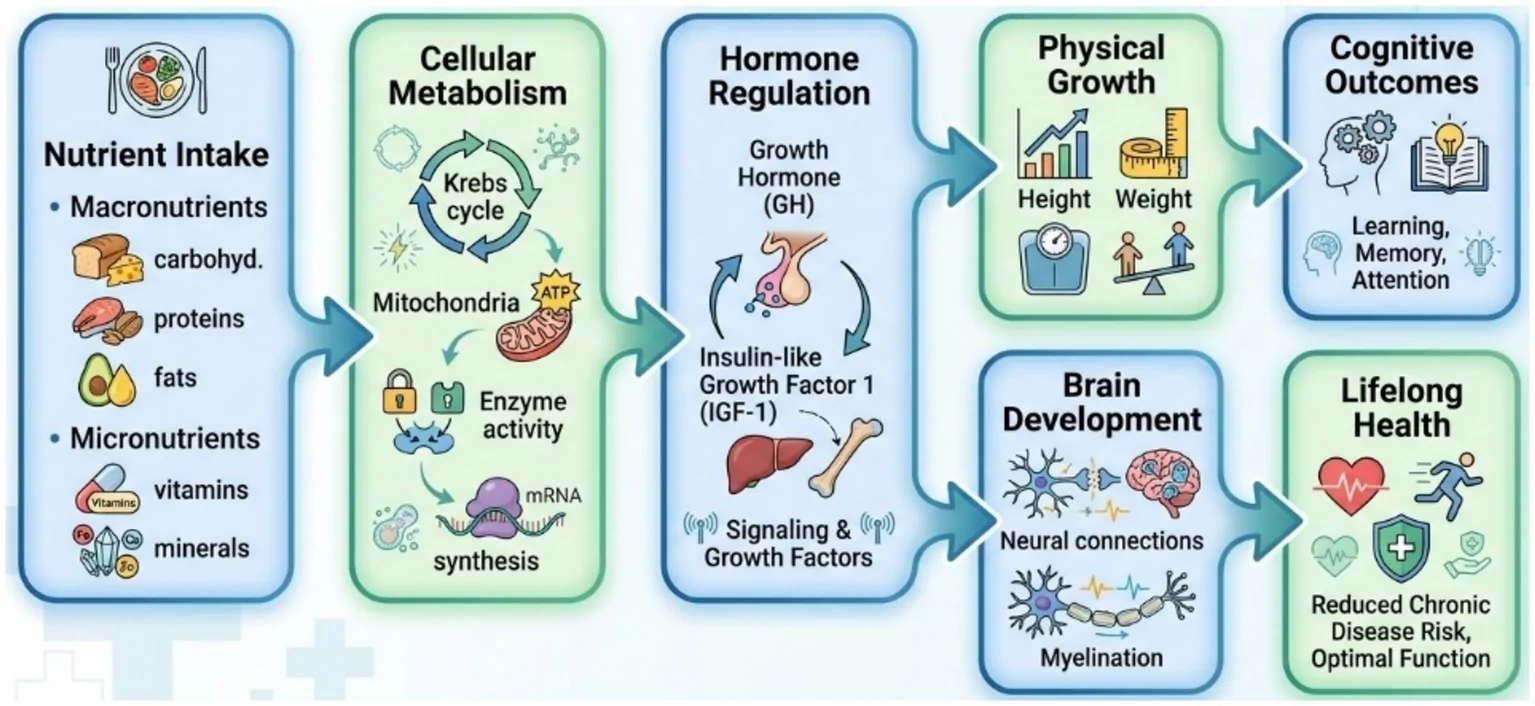
Mechanism linking pediatric nutrition to growth and development.

#### Cultural and economic determinants

Social and economic conditions greatly affect the nutrition of children during their early years. Children who come from poverty-stricken families are at much higher risk for undernutrition, micronutrient deficiency, and limited dietary variety since they typically have limited access to food that contains the nutrients they need. Nutritional inequities exist at a global level as well, with data indicating that children from the poorest socioeconomic level have a much greater incidence of stunting and wasting compared to those from wealthier socioeconomic levels (17). Food insecurity further compounds the risk of malnourishment by limiting a child’s access to adequate and a variety of foods. Food insecurity causes both malnourishment and unhealthy eating patterns and increases the risk of obesity and other health problems later in life (18).

The mother’s level of education, her parenting style, and the family unit structure can also greatly influence child nutrition. Mothers who are educated tend to breastfeed properly, provide their children with a variety of complementary foods, and obtain medical care for their children when necessary; therefore, children of educated mothers generally achieve better nutritional outcomes. Research conducted in South Asian countries highlights the essential role that mothers play in their children’s fulfillment of minimum dietary diversity standards. If they have had limited formal education, it is likely that their children will not achieve these standards. It emphasizes how important maternal knowledge and empowerment are to ensuring children receive sufficient nutrition (19). Additionally, the dynamics within households and limited maternal decision-making authority can negatively impact access to adequate nutrition and appropriate healthcare, perpetuating the risks associated with malnutrition (20).

Accessing health care and nutrition services is another major factor affecting childhood nutrition and child malnutrition. Services that prevent or treat malnutrition, such as growth monitoring, micronutrient supplementation, and nutrition counseling, are necessary to effectively identify and treat children who are malnourished; however, barriers to accessing health care exist across the rural and low socio-economic sectors of communities that lack the required support systems to facilitate the access of children to nutrition services (21). Through the integration of nutrition screening and nutrition support services with routine pediatric health care providers, children at risk for food insecurity can be identified and referred for appropriate services. Addressing these socio-economic and social determinants is vital in efforts to reduce child malnutrition and to enhance children’s overall health (22).

#### Environmental and cultural determinants

The nutritional outcomes of children are significantly influenced by their environment, especially regarding urban and rural differences. A common reason for the increased occurrence of malnutrition among children in rural communities is limited access to healthcare services, as well as the quality and availability of both transportation and market infrastructure. Additionally, rural communities generally do not have access to diverse, nutrient-dense foods. Due to the dependence of families on agricultural production and the seasonal availability of food products, families may also experience variable food intake due to variability in crop production and the price of food products (23). As a further example of this, rural areas are less likely than urban areas to have adequate food distribution systems because of poor physical infrastructure, such as poorly maintained roadways and limited food storage facilities. Urban areas are usually more accessible to healthcare services, restaurants, and supermarkets with more choices of foods; however, many of the same advantages enjoyed in urban settings by children are offset by the rapidly increasing procurement and consumption of highly processed foods that are high in refined sugars, sodium, and saturated fats. Consequently, children living in urban settings are now at a greater risk of becoming overweight or obese (24).

Environmental factors like sanitation, agricultural productivity, water quality, and climate change impact child nutrition in numerous ways. Aspects of poor sanitation practices and contaminated oral or non-potable water sources lead to a higher incidence of infectious diseases, e.g., diarrhea and intestinal infections, which result in less nutrient absorption, contributing to stunted growth in young children. Changes to the climate that occur, including extreme weather events, disrupt agricultural production or food supply chains, impacting the availability and affordability of nutritious foods (25). Vulnerable populations living in low-income areas experience the greatest impact from the challenges posed by these environmental factors; food insecurity and limited ability to adapt increase the nutritional challenges faced by them. In these situations of inadequate dietary intake combined with repeated infections, children are at risk for stunting, wasting, and micronutrient deficiencies (26).

Food consumption as well as patterns of feeding children are further affected by cultural, traditional, and religious factors, as well as the food systems of our communities (27). Certain cultural practices, such as eating a traditional diet consisting of culturally valued, religiously accepted, and locally obtained foods, can help to improve nutrition and health by promoting the use of a variety of foods produced locally. Additionally, cultural practices will determine how well mothers breastfeed, what complementary feeding practices they use, and what decisions are made by caregivers about infant and child feeding (28). However, various cultural beliefs and food taboos may limit the intake of, and thus the availability of, high-quality, high-nutrient foods, e.g., eggs, meat, and dairy, for women who are pregnant and their children during their first years of life, which may increase the risk of developing micronutrient deficiencies or becoming undernourished. Furthermore, the way that caregivers use their knowledge and understanding of food, family dynamics, and gender roles in the household all contribute to how food is distributed among the members of the household, and can disadvantage women and young children in certain cultural contexts (29).

There are various factors that determine children’s access to an adequate amount of nutrition; these include political and structural issues. Government regulations regarding the production of food, agricultural subsidies, food distribution, access to health care, and social protection programs all greatly impact the availability and affordability of food and nutrition. Poor governance, inadequate investment in food systems, and inadequate healthcare can compound the inequities of nutrition for marginalized and underserved groups (30). For example, a lack of effective regulation of food markets may produce a greater amount of processed and unhealthy foods. In contrast, a lack of investment in rural infrastructure may make it more difficult for individuals in rural communities to gain access to health care and adequately priced foods. To address these structural determinants of nutrition inequities, coordinated policy interventions are necessary to strengthen food systems, enhance access to health care, and create equitable resource distribution (31).

In order to improve nutrition among children, it is critical to develop culturally competent, policy-based approaches to support the complex interactions between cultural practices, political structures, and environmental conditions. Government, community, and health systems must work together to build sustainable food systems, strengthen infrastructure, support education regarding culturally appropriate nutrition, and provide equitable access to nutritious foods and health care for all children (32). By integrating environmental, cultural, and structural issues into nutrition policy and programming, it is possible to reduce the disparities in nutrition among children and develop environments that will allow all children to reach optimal growth, development, and health long-term (33).

#### Health consequences

Child nutrition inequities result in multiple health challenges, such as stunted growth, being under- or overweight, and micronutrient deficiencies like iron deficiency anemia. Prolonged food insecurity, as a result of poverty and a lack of access to a variety of healthy foods, greatly increases the risk of developing stunting, which is a reflection of ongoing under-nutrition and negatively impacts a child’s growth and development in general (34). Stunted children are shorter than expected for their age and generally have a weaker immune system; consequently, they will often be at greater risk for being affected by infections and illness. In low-income areas, the lack of dietary variety combined with inadequate amounts of essential nutrients frequently leads to conditions such as iron deficiency anemia, which results in reduced levels of hemoglobin in the blood and compromises the transport of oxygen throughout the body. This condition contributes to fatigue, and there is a decreased level of physical activity in those who suffer from anemia (35). Additionally, there is decreased cognitive ability as a result of being anemic that ultimately limits a child’s ability to properly grow, develop, and learn. Many low-income populations are experiencing a transition in their nutrition patterns; in other words, consuming foods that are highly processed, inexpensive, and energy-dense as opposed to consuming a wide variety of healthy foods and getting all the essential nutrients necessary for good health. Increased levels of childhood overweight and obesity have increasingly contributed to the “double burden of malnutrition,” due to the fact that urban and low-income environments often contain both undernutrition and obesity at the same time due to the lack of full-time access to healthy food sources (36).

The long-term effects of poor childhood nutrition have been established as significant factors affecting cognitive development, educational achievement, and long-term well-being beyond immediate physical health. Clinical and population-based studies have shown that children who do not get sufficient nutrition during critical growth periods are at a higher risk of delayed cognitive development, have decreased attention and memory capacity, and have more trouble learning. As a result of these challenges, children perform at lower academic levels, attend school less frequently, and receive less education (37). The effects of early-life malnutrition can persist into adulthood and significantly impact the productivity, employment, and socioeconomic status of an adult. Additionally, those who do not receive adequate nutrition in early childhood are more likely to develop non-communicable diseases in adulthood, such as heart disease, type 2 diabetes, and metabolic disorders. Early deficiencies in nutrition may also adversely affect metabolic programming and the development of the immune system and increase individuals’ susceptibility to chronic health conditions for the rest of their lives (38).

Childhood malnutrition not only influences the health of individual children; it also provides a link between generations of children suffering from both poor health and socio-economic disadvantage. Children born to mothers who suffered from malnutrition during their childhood or adolescence are at increased risk for complications during pregnancy and delivery, and their babies can have low birth weights or impaired growth. Those babies are born with a poor nutritional foundation that contributes to their vulnerability to disease, delayed development, and continued undernutrition as they grow (39). When combined, these factors result in a cycle of poverty, poor health, and lack of sufficient access to proper amounts of food, which continues across multiple generations. In order to break the cycle of malnutrition, it is necessary to have comprehensive strategies that will improve food quality, improve access to health care, and address the root causes of poverty and social inequality (40). If societies prioritize equitable access to health care and sufficient access to quality foods and education, it is possible to reduce the long-term negative impacts of malnutrition on a child’s health and well-being, while promoting healthy outcomes for all generations of children (41).

#### Nutritional support strategies

Successfully addressing malnutrition in childhood requires a coordinated and multi-level approach to action on the part of the governments, communities, healthcare systems, and families (42). There are many factors that influence a child’s nutrition status; therefore, an intervention targeted at just one factor typically does not provide the necessary support to improve nutrition outcomes. There are a number of strategies that can be taken at the policy level, e.g., national nutrition programs, food fortification, to create an environment that allows increased access to nutritious foods. Community-based initiatives, such as school feeding programs, support some of the neediest populations by encouraging healthy eating behaviors and by giving culturally appropriate information about child nutrition (43).

At the healthcare and household levels, there are many interventions that can help improve child nutrition. Routine checks for children’s health and growth monitoring, along with supplemental vitamins and minerals for children and nutrition counseling for families, will help identify and manage children who are at risk for malnutrition (44). Breastfeeding promotion, providing appropriate complementary foods, educating family caregivers about appropriate feeding practices, and other maternal behaviours at the household level will have a direct effect on children’s nutrition and health. The research supports the effectiveness of using integrated strategies that address all the determinants of nutrition in a holistic manner, or at least simultaneously, to improve child health and reduce the risk of disparity amongst different populations (45).

#### Policy-level interventions

Interventions on a policy level are key for addressing malnutrition in children at a population level because they work to target both the direct nutrition deficiencies of children as well as the social determinants of health. The implementation of national nutrition policies and strategies provides the framework for these interventions through setting priorities and guidelines for child nutrition programs (46). Countries that have implemented extensive child nutrition policies, such as standards for school meals, programs for supplementing food, and systems for tracking the progress and monitoring of programs, have seen improved growth and quality of child diets. This has been established through systematic reviews (47). In Afghanistan, policies aimed at improving the nutrition of mothers and children through prenatal supplementation, educational programs, and targeted support to vulnerable populations were associated with a reduction of low-birth-weight children from 18 to 13% within 5 years, demonstrating the effectiveness of a coordinated approach to nutrition policy (48).

Food fortification initiatives serve as a crucial method for policy-level action. Through the widespread utilization of iron, Vitamin A, and iodine fortification, many countries have made enormous progress in mitigating micronutrient deficiencies (33). For example, mandatory iodization of salt and Vitamin A food fortification have been shown to have reduced the prevalence of iodine-deficient disorders by 20–30% in school-aged children in a variety of low- and middle-income nations, while also reducing Vitamin A deficiency by up to 25% among children of preschool age (49). Additional efforts in the form of subsidies, cash transfers, and legislative measures also increase the accessibility of nutritious foods to promote equity. Conditional cash transfer programs have demonstrated success in increasing dietary diversity and decreasing stunting in children from low-income families (50).

Policy interventions have had limited success due to insufficient funding and a lack of enforceable regulations. There is no culturally appropriate method for most communities, especially those that are rural or marginalized. Therefore, national programs are improving nutritious food indicators overall, but the disparities that exist can also be related to inequitable access to healthcare, and to a large extent, not providing for the poorest and most disadvantaged children (51). An evaluation of these types of policies indicates that policies will achieve maximum benefit by combining them with monitoring systems, like enabling local authorities or families to monitor the latest account balances, and by using a variety of different agencies, i.e., implementing services through a partnership approach with local government agencies. All of these items provide the framework for successfully improving child nutrition on a large scale. Still, without appropriate government commitment and sufficient resources and coordination with community and health care to achieve a significant outcome, the impact will be minimal (52).

#### Community-based interventions

Local communities can be instrumental in addressing the nutritional needs of children through programs specifically designed for children, households, and other local environments where children live and grow. A common form of intervention is the school meal program, with the objective of providing school-aged children with nutritionally adequate meals that will assist them in achieving a more balanced diet through the provision of a greater quantity of daily nutrients such as energy, protein, vitamins, and minerals (53). The current evidence base shows that school meal programs are able to increase the number of nutrients children consume daily by 15–20%, as well as decrease rates of malnutrition and anemia in children. In addition to improving the nutrition status of school-aged children, school feeding also positively affects attendance at school, concentration in class, and academic performance (54).

In addition to school meal programs, community-based nutrition programs, e.g., counseling, education, and information dissemination, have resulted in significant changes in feeding practices at the household level and subsequent changes in growth outcomes for children. The community-based nutritional counseling programs in Nigeria resulted in an 18% increase in dietary diversity scores and increased fruit and vegetable intake (55). An example of this is the integrated community-based intervention program in rural Nepal, which used combined approaches, i.e., growth monitoring, caregiver education, and food supplementation. As a result, there was a 12% decrease in the prevalence of malnutrition among children under five. Non-governmental organizations (NGOs) and local initiatives are typically involved with community-based programs as they provide funding, training, and culturally relevant and community-specific programs (56).

Community-based interventions are successful in being both effective and flexible; however, there are still some challenges when it comes to their ability to be scaled up, sustained, and delivered consistently to all intended members of the target population. One such challenge could be the presence of a limited amount of resources, both human and material, to deliver the intervention; these limitations often lead to issues with accessing or delivering the program to the target audience. Community-focused approaches appear to have better success when they are used alongside supporting policies, ongoing monitoring, and continuous active participation from local stakeholders to help ensure that the interventions offered are culturally appropriate, equitable, and sustainable (57) (Table 2).

**Table 2:** Global trends and key outcomes of childhood malnutrition and nutrition-related conditions.

Type of Malnutrition/nutrition-related condition	Description	Global trend/defined outcome	References
Stunting	Low height-for-age, reflecting chronic undernutrition	Approximately 148 million children under 5 years were affected globally in 2022; prevalence has declined substantially since 2000 but remains high in low- and middle-income countries (LMICs)	(87)
Wasting	Low weight-for-height, reflecting acute undernutrition	Approximately 45 million children under 5 years were affected globally in 2022; wasting remains particularly prevalent in fragile and resource-limited settings	(87)
Underweight	Low weight-for-age	Global prevalence has declined since 2000, although underweight remains concentrated in South Asia and sub-Saharan Africa	(87)
Childhood overweight/obesity	Excess body weight relative to height/age	Approximately 37 million children under 5 years were overweight in 2022, with prevalence increasing in many regions	(87)
Iron deficiency	Inadequate iron status, potentially resulting in impaired erythropoiesis and anemia	Iron deficiency remains one of the most common micronutrient deficiencies globally and contributes substantially to childhood anemia	(88)
Vitamin A deficiency	Inadequate vitamin A status affecting vision and immune function	Remains a significant public-health concern in several LMICs, although supplementation programs have reduced deficiency-related morbidity in high-risk populations	(89)
Iodine deficiency	Inadequate iodine intake affecting thyroid hormone production and neurodevelopment	Population-level iodine status has improved substantially following salt-iodization programs, but deficiency persists in some vulnerable populations	(90)
Zinc deficiency	Inadequate zinc intake or status affecting growth and immune function	Remains common in populations with low dietary diversity and high reliance on plant-based staple foods	(91)
Protein-energy malnutrition	Deficiency of energy and protein sufficient to impair growth and physiological function	Severe forms remain concentrated in areas affected by poverty, food insecurity, infection, conflict, and humanitarian crises	(92)
Hidden Hunger	Deficiencies of essential vitamins and minerals despite adequate or excessive energy intake	Remains widespread globally and may coexist with overweight and obesity, particularly where diets are energy-dense but nutrient-poor	(91)
Double burden of malnutrition	Coexistence of undernutrition and overweight/obesity or micronutrient deficiencies	Increasingly observed within the same households and populations, particularly during nutritional and epidemiological transitions	(89)
Rural malnutrition	Malnutrition associated with limited food, healthcare, sanitation, and nutrition-service access	Rural children in many LMICs experience higher risks of stunting, wasting, and dietary inadequacy than children in more advantaged settings	(93)
Urban malnutrition	Malnutrition associated with urban poverty, food environments, and poor dietary quality	Rapid urbanization is associated with changing diets, increased consumption of ultra-processed foods, and rising childhood overweight alongside persistent undernutrition	(86)
Food insecurity	Inadequate or uncertain access to sufficient, safe, and nutritious food	Remains a major global determinant of child undernutrition and is associated with poorer dietary diversity and increased risk of stunting and wasting	(90)
Low birth weight	Birth weight below 2,500 g	Approximately 19.8% of live births globally were estimated to have low birth weight in 2020, with the greatest burden in South Asia and sub-Saharan Africa	(94)
Childhood Anemia	Reduced hemoglobin concentration, frequently related to iron deficiency	Anemia remains highly prevalent among young children globally and is associated with impaired cognitive and physical development	(92)
Dietary imbalance	Inadequate dietary diversity or inappropriate proportions of essential nutrients	Poor dietary diversity remains common among young children in LMICs and is associated with inadequate intake of essential micronutrients	(89)
Fast-food/ultra-processed food consumption	Frequent intake of energy-dense, nutrient-poor foods	Consumption is increasing in many urban and transitioning populations and is associated with higher energy intake and increased risk of childhood overweight	(88)
Breastfeeding gaps	Suboptimal initiation, exclusivity, or duration of breastfeeding	Global exclusive breastfeeding among infants younger than 6 months remains below the recommended 50% target, despite gradual improvement	(95)
Climate-related nutrition risk	Nutrition effects resulting from climate shocks, food-system disruption, and reduced food availability	Climate change increasingly threatens food security and dietary quality and may increase risks of childhood undernutrition, particularly in vulnerable populations	(96)

#### Healthcare system-based interventions

Negative health effects on children can be managed through a variety of interventions based on early detection of malnutrition, Prevention of malnutrition, and management of malnutrition by healthcare systems. Healthcare systems can help identify children who are at risk of becoming malnourished or developing micronutrient deficiencies by providing services such as growth monitoring and routine pediatric visits. By measuring an individual’s height, weight, and body mass index (BMI) and tracking those measures over time, providers can identify if individuals are moving away from healthy growth patterns and intervene before an individual develops serious complications (58). Growth monitoring allows healthcare providers to detect malnutrition early, making it possible to provide referrals to nutrition support services, dietary counseling, or medical treatment in a timely manner. The integrated primary care services that provide nutrition services also offer preventive services such as immunizations, nutrition screening, and supplementary programming, all of which can ultimately improve an individual’s nutrition status or outcome. Children from low-income families often have limited access to nutritious food and healthcare services; thus, these interventions are critical for them (59).

Besides the regular monitoring of children’s micronutrient levels by healthcare systems and agencies, supplementation and nutrition support programs are often developed to reduce the prevalence of common deficiencies. These programs typically provide iron, vitamin D, and multivitamins to pregnant women through various channels of maternal-child healthcare services, and have been extremely successful in helping to eliminate nutrient deficiencies and to promote healthy growth and development in children (60). Vulnerable populations, in particular, will benefit from the support provided through these programs because they are at an increased risk of developing anemia, having impaired immune systems, or delayed development due to an inadequate diet. Healthcare interventions also provide healthcare providers with the opportunity to identify and manage metabolic risk factors for high-risk populations. These improved health outcomes, in turn, create an environment where integrated healthcare interventions can yield inter-generational benefits by contributing to improved long-term health outcomes for subsequent generations by improving maternal nutrition during pregnancy (61).

Primary care facilities provide nutrition counseling, which enhances the benefits of these services by helping caregivers utilize better feeding practices. The purpose of individual counseling sessions is to educate caregivers on the value of exclusive breastfeeding, introducing appropriate complementary foods when the child has reached the required development, and ensuring a variety of foods are fed throughout infancy and childhood. Healthcare providers may also promote healthy behaviors related to establishing balanced diets and engaging in regular physical activity. These behaviors are key factors in reducing childhood obesity and developing chronic diseases related to overweight status (62). By incorporating education into clinical care models, healthcare practitioners can assist caregivers in making the best decision regarding the long-term health of their child. Even with many successful healthcare system-based nutrition interventions, there are still many obstacles to participation by families, including, but not limited to, lack of time, cultural beliefs, lack of transportation, and lack of understanding of how health interventions work. Rural families and families living in poor areas may also have limited access to healthcare resources and trained healthcare workers, resulting in disparities in the availability and utilization of nutrition services (63). Overall, developing culturally competent programs, flexible delivery models such as mobile clinics or community health workers, and increasing collaboration between healthcare systems and local communities are important parts of overcoming these obstacles. In addition, ongoing monitoring and evaluation will ensure that the interventions continue to be effective and relevant to diverse populations. According to the evidence, healthcare-based nutrition interventions work best when implemented together with community-based efforts and supportive public policies to create a comprehensive solution that addresses not only the immediate nutrition needs of children but also the underlying social conditions that affect child health (64).

#### Household and individual-level interventions

Nutrition programs at home and the family level aim to provide caregivers with the information, expertise, and support needed to improve children’s food offerings. Caregivers are essential in deciding what, how often, and when a child eats. For this reason, increasing caregiver capability to provide adequate nutrition becomes a major part of achieving good health for young children. Multiple studies have demonstrated that breastfeeding promotion programs are the best way to enhance infant feeding habits and prevent poor nutrition during infancy (35). Breast milk contains several nutrients, immune boosters, and bioactive compounds that help strengthen the immune system and stimulate healthy growth in infants. In low-income countries, peer counseling, home visits from trained community health workers, and mother-to-mother support groups have similar positive impacts on increasing the rates of exclusive breastfeeding by approximately 15 to 25%, increasing infant health, and reducing the risk of infections and growth retardation (65). Caregiver education on complementary feeding assists the caregiver in introducing age-appropriate, nutrient-dense foods to children when breastfeeding is no longer adequate. Family caregivers receive education, training, and skill-building through nutritional education sessions and demonstrations to better understand how to prepare a variety of meals using local foods. As a result of participation in these programs, children experience improved nutritional adequacy from a variety of foods; improved food variety; and increased growth, which helps them grow and develop during the important one-to-three-year-old period (66).

Family caregiver education and empowerment strategies provide an additional avenue to encourage long-term healthy eating among families. Family nutrition education programs and social support systems that have been designed to assist families in identifying sources of nutrition education include, but are not limited to, community-based programs similar to the Special Supplemental Nutrition Program for Women, Infants, and Children (WIC), which have been shown to improve dietary habits among both children and their caregivers (67). Family nutrition education and support programs may provide nutritional knowledge, food assistance, and behavioral interventions to reinforce improved dietary habits, thereby decreasing the risk of developing unhealthy dietary habits. Family-centered obesity prevention strategies targeting children aged 0–4 years have been shown to increase the frequency of healthy dietary practices by 7 to 10 percent. Long-term multidimensional programs, such as the WIC program, have increased the number of low-income families adhering to recommended dietary guidelines and reduced the prevalence of unhealthy eating practices among low-income families. Interventions will achieve greater success when they can effectively adapt to the local culture, for example, respect for traditional food preferences, and to the social and economic realities of caregivers. In general, programs that include caregivers in meaningful ways during the decision-making process and that focus on cultural preferences have a better chance of resulting in longer-term behavior change (37). Barriers to maximizing the effectiveness of these interventions include: maintaining long-term compliance of participants, expanding successful pilots into the broader community, and securing ongoing financial resources. Nevertheless, household and individual-level interventions are an important aspect of a comprehensive child nutrition strategy. When used in combination with community-based programs, health-care services, and supportive policies, household- and individual-level interventions can help develop knowledge of caregivers, improve feeding practices at home, and ultimately enhance the physical growth, development, and health of children (68) (Figure 2).

**Figure 2:**
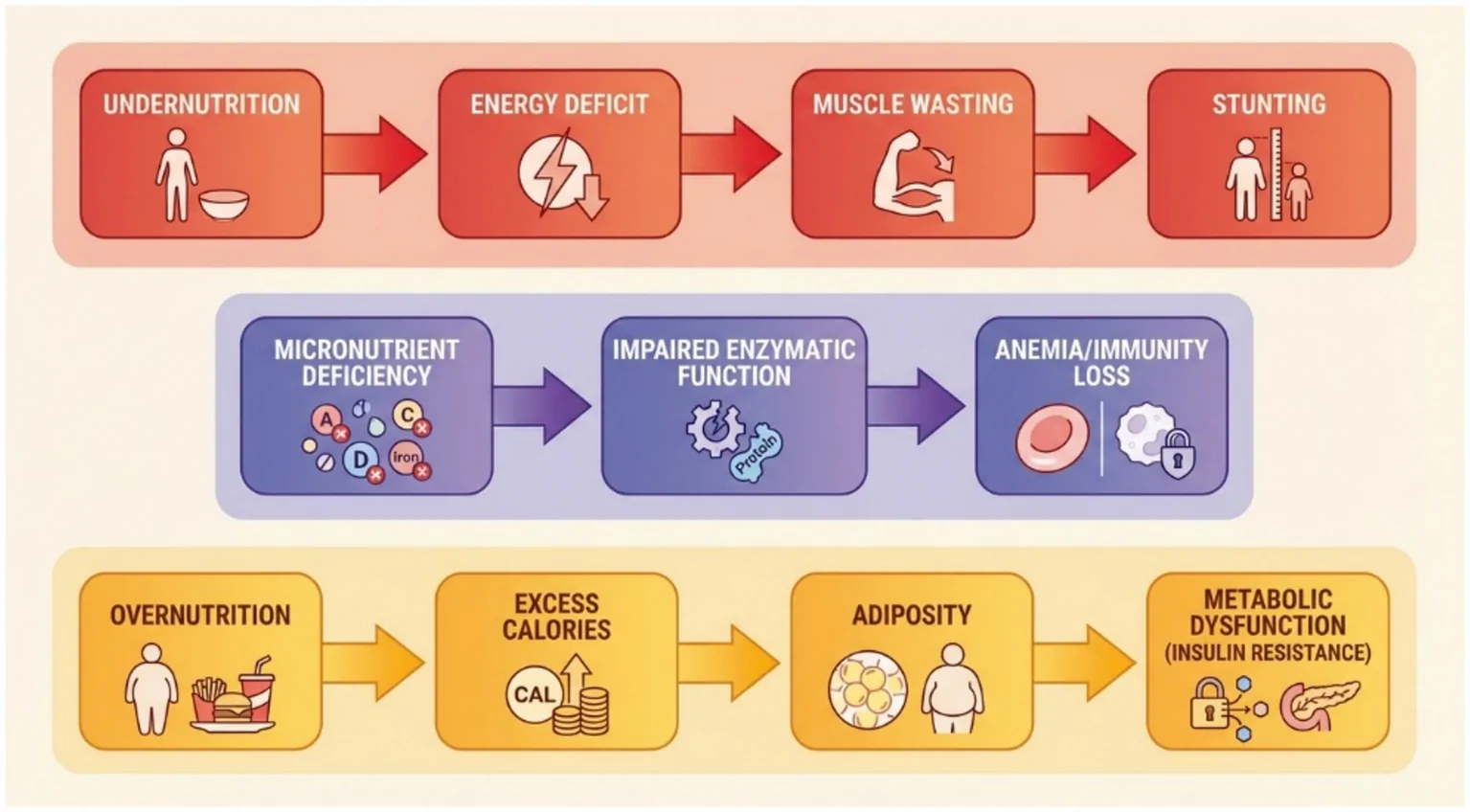
Pathophysiological pathway of malnutrition.

#### Evidence of impact on health inequalities

Research demonstrates that child health can be greatly enhanced using nutrition interventions aimed at multiple levels. Examples include school meal programs, promotion of breastfeeding, and community nutrition programs, all of which have been measured to have an impact. Results from a school lunch reform in Sweden have shown a 7% increase in adult height and a 9% increase in educational achievement among children from low-income households, which illustrates one long-term impact of improving equitable access to nutritious foods and meals (69). Exclusive breastfeeding interventions have also provided reduced morbidity and improved growth in high-risk populations, with rates of exclusive breastfeeding increasing at a rate of 15–20% when targeted programs were implemented towards this population (70).

To help the most disadvantaged children achieve optimal health, efforts must take place at the level of the policy, the whole community, and the home, and all of the strategies to help must be integrated. For example, community programs targeting food insecurity and improving the availability of healthy foods in less healthy neighborhoods have improved access to high-quality food and increased the number of vitamins and minerals consumed by low-income families (71). Nevertheless, there are still gaps in service provision; for instance, many interventions are not successfully reaching rural, conflict-affected, or informal urban populations; service availability may also be hampered by limited resources and inadequate culturally appropriate programs. Successful interventions have taken a multi-sectoral approach to coordinating support for those affected by food insecurity, providing culturally appropriate programming, and monitoring recipient progress continuously (3). Barriers to the provision of successful, equitable interventions include inequitable distribution, insufficient funding, and a lack of sustainable support for long-term implementation of socially equitable health solutions. In sum, there is evidence to suggest that if designed appropriately, social justice-focused interventions can lead to improvements in the health status of disadvantaged populations; however, there are ongoing structural and contextual barriers to the effective implementation of such interventions (72) (Table 3).

**Table 3:** Social and economic determinants of nutritional inequality and research-based nutritional outcomes.

Determinant	Description	Research-based nutritional outcome	References
Poverty	Limited household financial resources and purchasing power	Higher household poverty is associated with reduced dietary diversity and increased risks of childhood stunting, wasting, and underweight	(97)
Income inequality	Unequal distribution of household and community resources	Greater socioeconomic inequality is associated with disparities in child growth and nutritional status, particularly among disadvantaged households	(97)
Maternal education	Maternal knowledge, literacy, and education related to childcare and feeding	Higher maternal education is associated with improved complementary-feeding practices, greater dietary diversity, and lower risk of child undernutrition	(98)
Employment status	Household employment and income stability	Stable household employment and income are associated with improved food security and greater ability to obtain diverse and nutritious foods	(87)
Food prices	Cost and affordability of nutritious foods	Higher prices of nutrient-dense foods are associated with reduced affordability, lower dietary diversity, and poorer diet quality among low-income households	(88)
Household size	Number of household members sharing available resources	Larger households with limited resources may experience reduced per-capita food availability and increased risk of inadequate dietary intake	(98)
Gender inequality	Unequal access to food, healthcare, education, and household resources	Gender-related inequalities can contribute to differences in dietary intake, healthcare access, and nutritional outcomes among children, particularly girls in disadvantaged settings	(99)
Healthcare access	Availability, affordability, and utilization of preventive and curative health services	Limited healthcare access is associated with delayed identification and treatment of growth faltering, infections, micronutrient deficiencies, and acute malnutrition	(100)
Sanitation	Access to hygienic sanitation facilities and safe waste disposal	Poor sanitation increases exposure to enteric pathogens and is associated with recurrent infection and impaired child growth	(101)
Water Access	Availability of safe and reliable drinking water	Limited access to safe water is associated with increased infectious disease exposure and greater risk of childhood undernutrition	(102)
Social protection	Cash transfers, food assistance, vouchers, and nutrition-support programs	Social-protection interventions can improve household food security, dietary intake, and selected child nutritional outcomes, with effects varying by program design and context	(97)
Urbanization	Population movement toward urban areas and changes in food environments	Rapid urbanization is associated with simultaneous risks of persistent undernutrition and increased consumption of energy-dense, ultra-processed foods and childhood overweight	(85)
Education systems	Access to formal education and nutrition education programs	School- and community-based nutrition education can improve nutrition knowledge, dietary practices, and selected health behaviors among children and caregivers	(103)
Cultural norms	Cultural, traditional, and religious beliefs influencing food and feeding practices	Cultural feeding practices can influence breastfeeding, complementary feeding, food choice, dietary diversity, and acceptance of nutrition interventions	(104)
Migration	Population displacement, mobility, and disruption of social support systems	Migrant and displaced children may experience increased food insecurity, reduced healthcare access, and higher vulnerability to acute and chronic malnutrition	(105)
Conflict	Disruption of food production, markets, healthcare, and social services	Conflict-affected populations experience substantially increased risks of food insecurity, wasting, micronutrient deficiencies, and other forms of acute malnutrition	(104)
Agricultural access	Household and community access to agricultural production and food resources	Greater access to agricultural resources can improve household availability of diverse foods, although effects on child nutritional outcomes depend on income, food allocation, and caregiving practices	(100)
Market Access	Physical and economic access to food markets	Poor market access limits availability and affordability of diverse foods and is associated with lower household dietary diversity	(99)
Family dynamics	Caregiving arrangements, decision-making, and intra-household food allocation	Supportive caregiving and appropriate household decision-making are associated with improved breastfeeding, complementary feeding, and child dietary practices	(75)
Policy environment	Government nutrition policies, regulations, and implementation capacity	Strong nutrition policies and effective program implementation can improve coverage of nutrition services and contribute to reductions in child undernutrition and nutritional inequalities	(60)

#### Case studies and best practices

From evidence-based and integrated program experiences in both low-, middle-, and high-income countries, there are many useful lessons to be learned in terms of improving child nutrition. Some of the success stories include: In a high-income country, nutrition-focused pediatric quality improvement programs resulted in a 12% increase in adherence to dietary guidelines and improved growth outcomes for children receiving continuous care (73). In a middle-income country, community- and school-based integrated programs in Pakistan and India maintained nutrition services during the COVID-19 pandemic; without this, they would have contributed to a projected 15–20% increase in child malnutrition (74). In low-income countries, combined educator training, feeding support, and growth monitoring initiatives for children with developmental disabilities in Uganda and Bangladesh improved both developmental and nutritional status by 10–15% within 1 year of interventions (56).

The comparative analyses of the various programs reveal that multi-level approaches linking healthcare, community, and household programs are associated with the greatest success long term. Cultural adaptations, policy integration, and involvement of local stakeholders are all key factors for success and increase both the ability to replicate and sustain efforts. Lessons learned include that scalable programs require sustained funding, monitoring, and coordination with other sectors to achieve their full potential for impact. Finally, high-quality program design combined with evidence-based approaches provides the foundation for programs to be adaptable across diverse socioeconomic and geographic contexts while addressing the inequities associated with child malnutrition (57).

Research shows that eliminating the difference in nutrition for children is best accomplished with a multi-faceted approach at the different levels of intervention, i.e., government, community, health care systems, and home. Using a multi-level and cross-cutting approach allows for both addressing immediate nutritional needs at the child’s level and addressing the larger context of social determinants of child nutrition. Policies create a conducive environment for programs that deliver nutrition services to children (75). Policies at the national level, such as national nutrition strategies, food fortification, and social security policies, allow for community and home-level programs to provide effective delivery of nutritional services to children. These types of programs improve food security and dietary diversity for low-income families so that children have access to nutritious foods and health care. When policies are in place, there is an ongoing source of funding and an ongoing structure for nutrition-related programs at the community and home (58) (Figure 3).

**Figure 3:**
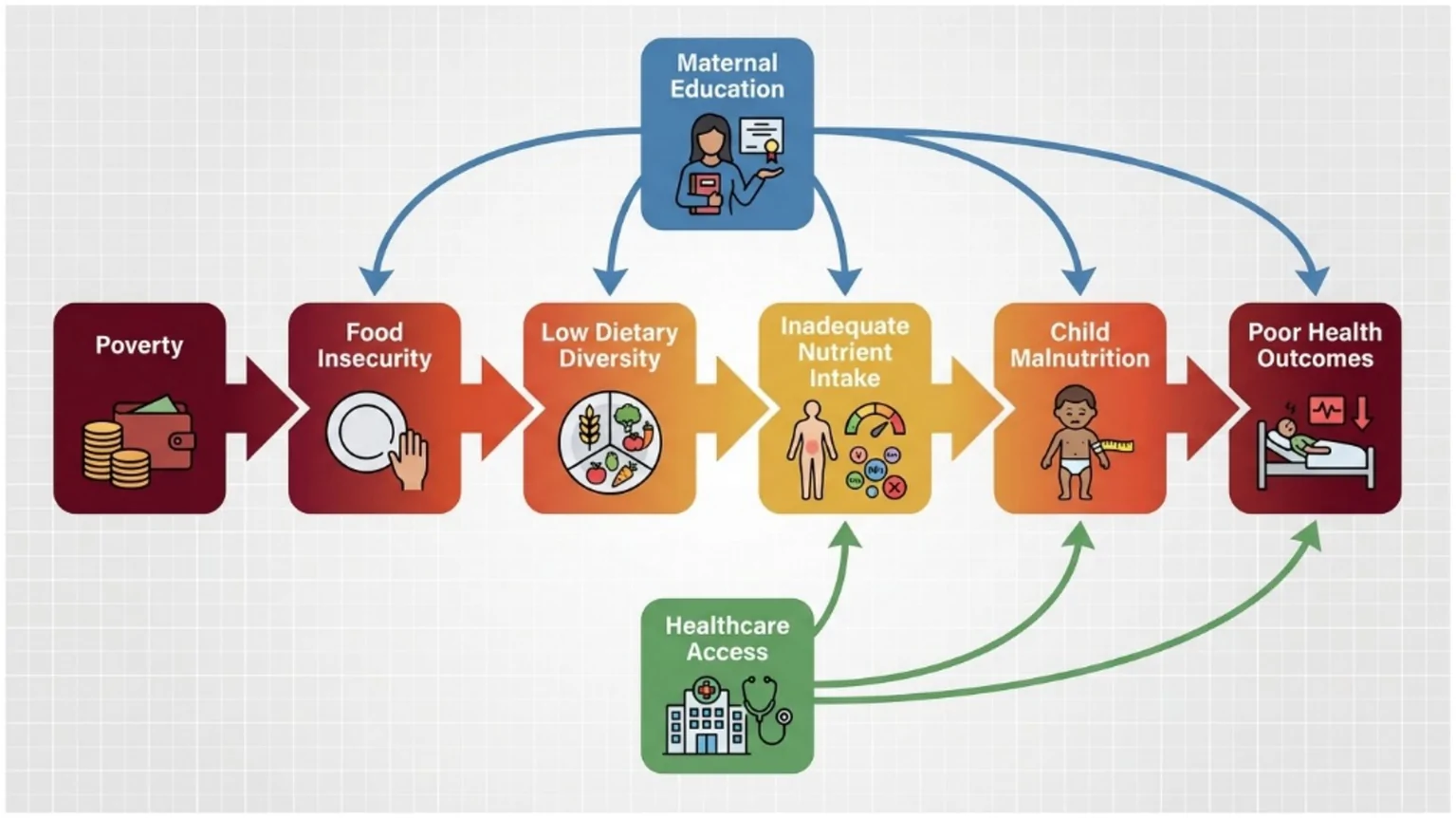
Socioeconomic determinants to nutritional outcomes.

At the local level, programs like food assistance programs in schools, education campaigns for good nutrition, home visitor programs, and volunteer-run programs are essential to helping fill the void created by national policies (12). All of these programs are especially helpful for families in low-resource and culturally diverse communities because in those communities, centralized programs typically do not adequately meet the needs of the most vulnerable members of society. Food assistance programs in schools help improve children’s diets and provide an incentive for students to attend school, and student cognitive function improves as a result. Home visiting and caregiver education programs help families with practical information on how to feed their children, how to maintain good hygiene, and how to care for their children. Community-based programs have a better chance of adapting their programs to the local culture, which enhances the likelihood that people will accept and use these programs. Additionally, partner organizations and community leaders enhance program sustainability through promoting local ownership of and participation in the program (60).

Community-level health improvement programs like school food assistance programs, educational campaigns promoting nutrition, programs providing home visitor services, and programs run by volunteers offer vital support to meet the local needs of the community that the federal government fails to meet through broader policies. These programs play an especially important role in providing health services for families living in low-resourced areas and families from different cultures (27). In fact, most community and family needs are not met by large-scale, central programs, and most of the people in the community with the greatest needs do not receive adequate support. Food assistance programs in schools are beneficial for both the child being fed and for providing an incentive for attending school; children being fed through school-based food assistance programs demonstrate improved cognitive abilities as a result (61). Home visitation and education programs for caregivers help families by providing practical information to enable them to feed their children, maintain good hygiene for their children, and care for their children. Community-based programs are likely to successfully adapt to the local culture of the community they serve, which increases the likelihood that community residents will use and accept them; additionally, collaborative organizations and community leaders increase program sustainability by helping to promote local ownership of and participation in program efforts (62) (Figure 4).

**Figure 4:**
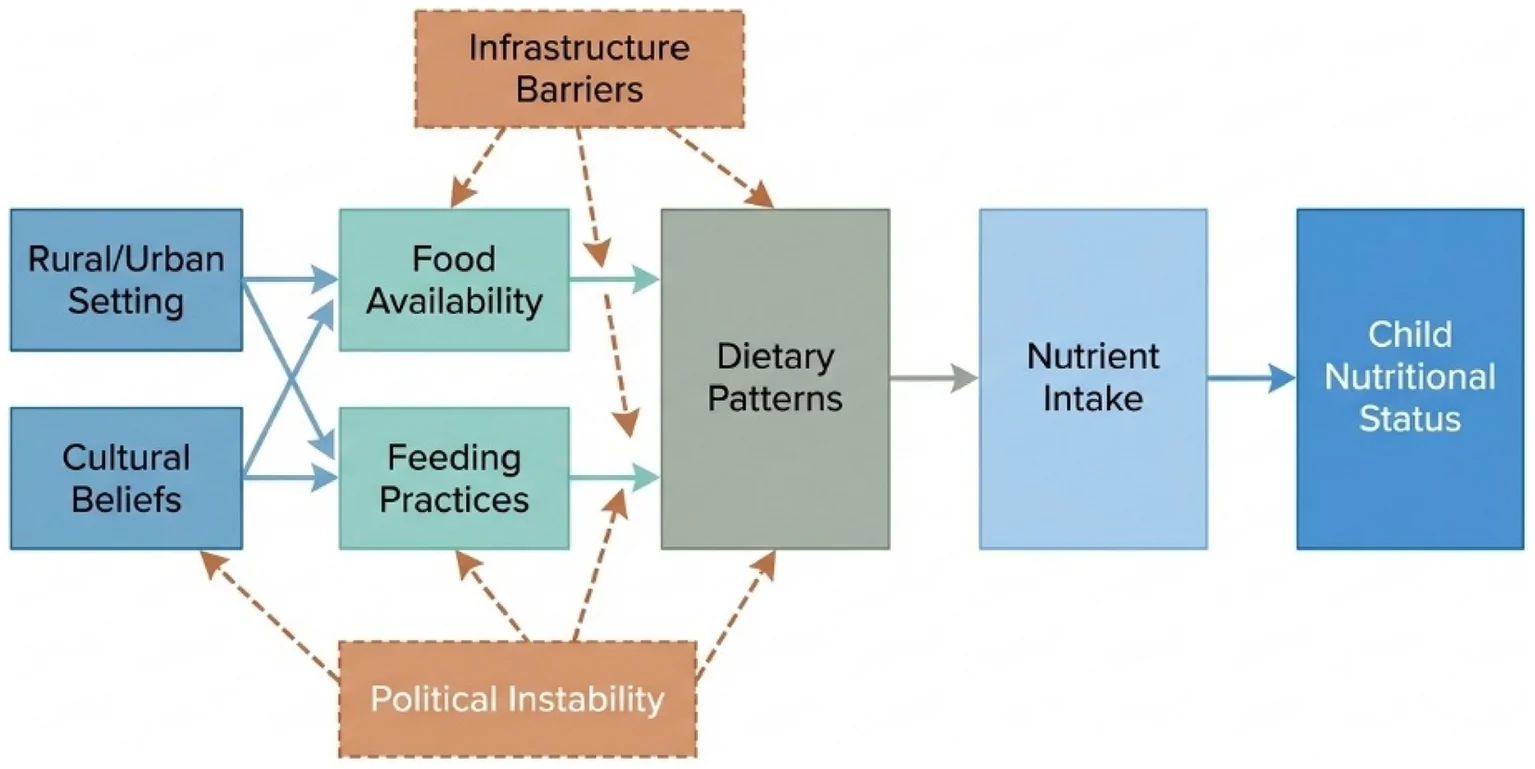
Environmental and cultural influence mechanism.

While there has been a lot of progress made using integrated strategies, there are still many gaps that limit the effectiveness of most child nutrition projects. For example, in many parts of the world, vulnerable populations—such as children living in conflict-affected areas or those living in remote rural or informal urban settlements—are often excluded from basic services. Poverty, geographic isolation, weak infrastructure, and lack of access to health care can cause these populations to lack access to basic services (63).

In addition to challenges caused by geography, there are also many cultural barriers to participation in nutrition projects and adherence to recommended nutrition interventions. Some of these barriers include the notion that different forms of feeding are appropriate depending on the gender of the child being fed; gender inequality within a family; perceptions around the poverty of the caretaker; and the education level of the caretaker (21). Moreover, many of the large-scale nutrition projects are designed to be funded only through short-term funding or through external donor support. Most of the traditional nutrition projects that were designed to address only undernutrition were not designed to adequately meet the ever-growing need to provide more support to children suffering from obesity or chronic diseases related to diet. As the landscape of nutrition continues to evolve, it is important to develop more comprehensive and flexible approaches that provide support to both malnutrition and obesity while also encouraging healthy eating and healthy living (64).

Future child nutrition programs must include multisectoral collaboration, cultural appropriateness, equity-based design, and sustainable financing to produce a meaningful and lasting effect at the population level. The implementation of child nutrition interventions requires strong partnerships between the public and private sectors, i.e., government agencies, healthcare systems, community-based organizations, and global partners. It must be integrated into larger development agendas that address poverty, education, food systems, and healthcare access. Continuous monitoring and evaluation mechanisms must be available to assess the effectiveness of individual child nutrition programs and to identify areas where implementation is lacking so that data-driven changes can be made to further enhance program fidelity (9). In addition to the need for multisectoral collaboration, culturally appropriate, equity-based design, and sustainable financing as part of any successful child nutrition program, promoting equity through inclusive and targeted strategies is critical to eliminating persistent disparities in the Nutrition of children globally. By aligning individual nutrition interventions with large-scale legislative and social initiatives that consider the underlying causes of health, societies can end the intergenerational cycle of malnutrition and allow for equal opportunities for optimal growth, cognitive development, and healthy lives for all children (65) (Table 4; Figure 5).

**Table 4:** Evidence of impact, geographic context, and program effectiveness of child nutrition interventions.

Intervention	Setting/population	Research-based observed impact	Key limitation	References
Food fortification	LMICs and populations with micronutrient deficiencies	Fortification programs have reduced population-level micronutrient deficiencies, particularly iron, iodine, folate, and vitamin A deficiencies, depending on coverage and fortification vehicle	Incomplete population coverage, inconsistent consumption, and variable fortification quality	(100)
School feeding	Primary-school children, particularly in food-insecure communities	School feeding has been associated with improvements in school attendance, dietary intake, food security, and selected anthropometric outcomes	Requires sustained funding, food supply, infrastructure, and program monitoring	(101)
Cash transfers	Low-income and food-insecure households in LMICs	Cash-transfer programs can increase household food expenditure, dietary diversity, and food security; some programs also improve child growth-related outcomes	Effects depend on transfer size, targeting, market conditions, and household use of resources	(102)
Breastfeeding programs	Mothers and infants, particularly in LMICs	Breastfeeding promotion increases early initiation and exclusive breastfeeding and is associated with lower risk of childhood infections and mortality	Cultural practices, inadequate counseling, workplace barriers, and limited health-system support may reduce uptake	(103)
Micronutrient supplementation	Children at high risk of micronutrient deficiencies	Vitamin A and other micronutrient supplementation programs can reduce deficiency-related morbidity and improve micronutrient status in targeted populations	Adherence, delivery coverage, supply interruptions, and program reach can limit effectiveness	(104)
Growth monitoring	Infants and young children attending primary healthcare services	Routine monitoring can identify growth faltering and acute malnutrition earlier, enabling referral and nutritional intervention	Inconsistent measurement, incomplete follow-up, and variable healthcare coverage can reduce effectiveness	(104)
Nutrition education	Caregivers, mothers, schoolchildren, and community populations	Nutrition education can improve caregiver knowledge, complementary-feeding practices, dietary diversity, and selected feeding behaviors	Knowledge gains do not always translate into sustained behavioral or nutritional changes	(105)
Community outreach	Rural and underserved communities	Community health and nutrition workers can improve screening, referral, breastfeeding support, and access to nutrition services	Workforce shortages, supervision requirements, transportation barriers, and limited scalability	(106)
Policy reforms	National and regional populations	Nutrition-sensitive policies, food regulations, social protection, and maternal-child health policies can increase intervention coverage and improve population nutritional outcomes	Political priorities, implementation capacity, financing, and intersectoral coordination influence effectiveness	(107)
NGO programs	Humanitarian, rural, and underserved populations	NGO-led programs can improve access to therapeutic foods, micronutrient supplementation, nutrition screening, and targeted household support	Dependence on external funding and variable continuity after program completion	(108)
Rural nutrition programs	Rural communities in LMICs	Community-based interventions can improve access to screening, supplementation, breastfeeding support, and complementary-feeding services and may reduce nutritional disparities	Geographic isolation, transportation difficulties, limited health workforce, and poor infrastructure	(109)
Urban nutrition programs	Children and families in urban and peri-urban communities	Urban interventions targeting diet quality and physical activity can improve nutrition knowledge and may reduce unhealthy dietary behaviors and obesity risk	Food environments, marketing of energy-dense foods, and socioeconomic inequalities may limit sustained effects	(110)
Digital nutrition campaigns	Adolescents, caregivers, and digitally connected populations	Digital interventions can increase exposure to nutrition information and may improve knowledge and selected dietary behaviors	Digital access, literacy, internet connectivity, and unequal technology availability limit reach in disadvantaged communities	(111)
Integrated care	Children receiving combined nutrition and healthcare services	Integrated nutrition-health interventions can improve identification and treatment of malnutrition while simultaneously addressing infections and other health conditions	Requires coordination among healthcare, nutrition, and community services	(112)
Maternal education	Mothers and caregivers of young children	Maternal nutrition and child-feeding education is associated with improved breastfeeding, complementary feeding, dietary diversity, and selected child nutritional outcomes	Limited literacy, time constraints, social norms, and limited access to education can affect implementation	(113)
Food subsidies	Low-income households and food-insecure populations	Subsidies targeting nutritious foods can improve affordability and increase access to selected nutrient-dense foods	Fiscal constraints, targeting errors, market responses, and sustainability may limit long-term effectiveness	(114)
Routine health checkups	Infants and children receiving primary healthcare	Regular child health visits facilitate early identification of growth faltering, anemia, micronutrient deficiencies, and feeding problems, allowing earlier intervention	Missed appointments, inadequate staffing, and unequal healthcare coverage can reduce program reach	(115)
Cultural adaptation	Communities with culturally specific dietary and feeding practices	Culturally adapted interventions can improve caregiver acceptance, participation, and adherence to recommended feeding practices	Requires local assessment, community engagement, and adaptation without compromising evidence-based recommendations	(116)
Multi-sectoral approach	Communities and populations affected by multiple nutritional determinants	Combining nutrition with health, agriculture, water and sanitation, education, and social-protection interventions can address multiple determinants simultaneously and improve food security and child health outcomes	More complex implementation, greater resource requirements, and challenges in intersectoral coordination	(117)
Long-term nutrition programs	High-risk populations and communities with persistent nutritional inequalities	Sustained interventions can improve continuity of nutrition services and support longer-term changes in dietary practices, food security, and nutritional status	Benefits may take years to become measurable and require stable financing and institutional commitment	(118, 119)

**Figure 5:**
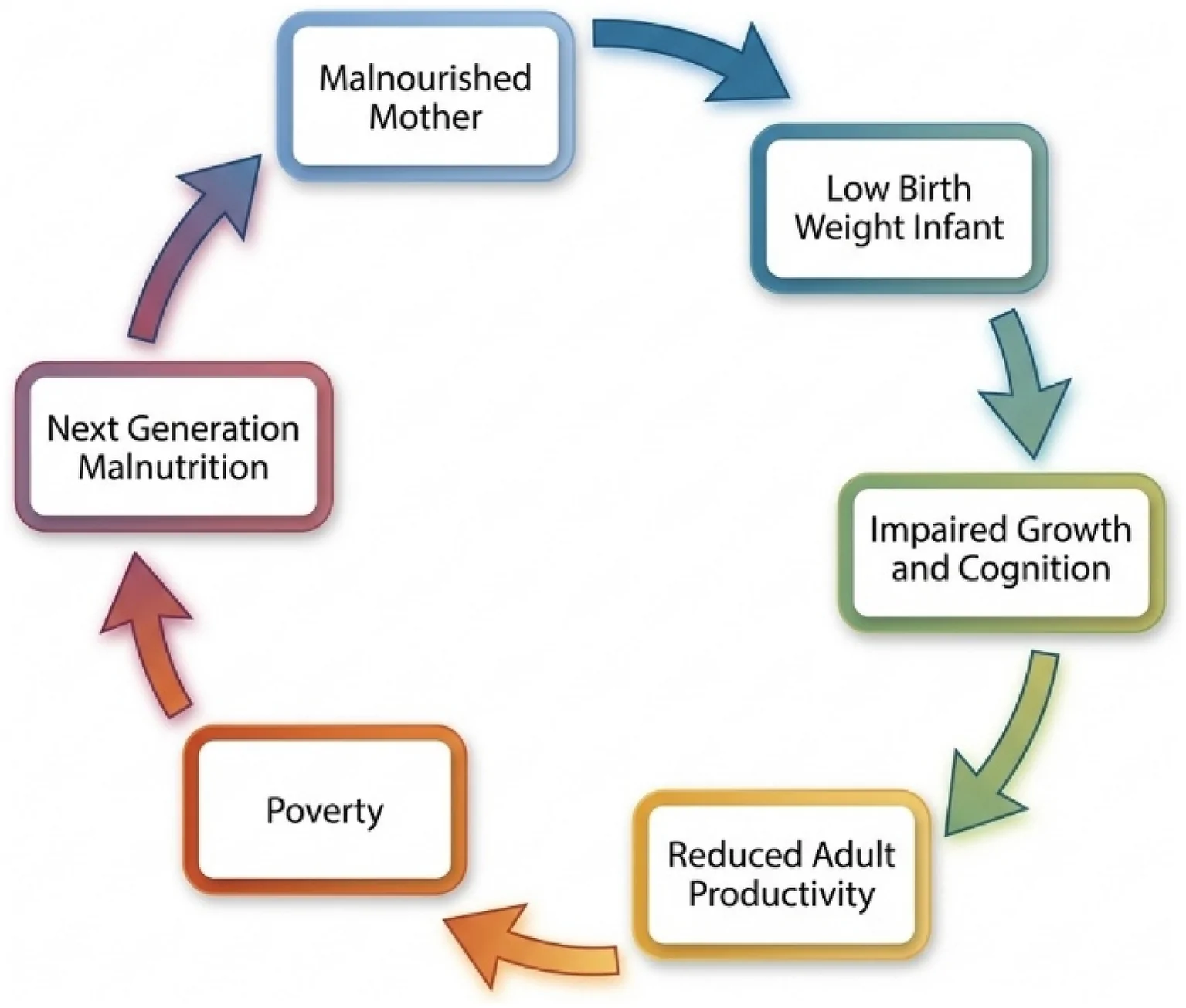
Intergenerational cycle of malnutrition.

#### Future directions and recommendations

Future action strategies in pediatric nutrition should center on addressing inequities and sustainability while providing focused provision for populations and regions with long-standing, persistent malnutrition and limited resources. Research should evaluate the long-term efficacy of multi-level interventions that are wrapped together in an integrated way, as well as develop culturally relevant, context-driven solutions that simultaneously address undernutrition as well as the emerging dual burden of malnutrition, of which obesity is one example. Innovative approaches using technology-assisted interventions, including, but not limited to, i.e., mobile health platforms, digital monitoring systems, and personal nutrition counseling, could improve program reach, improve caregiver engagement, and increase adherence to nutrition recommendations, specifically in remote or resource-limited settings. Programs and policies should be supported with comprehensive monitoring and evaluation systems that permit data-driven adjustments to programs, continual improvement within programs, and the ability to implement scalable interventions (66).

Strategic actions regarding the future direction in pediatric nutrition should involve solutions to address issues of equity and sustainability, with a specific focus on providing adequate nutrition for historically undernourished and disadvantaged populations, who have very few resources. Future research should assess whether there is a long-term effect of different levels of intervention when brought together in an integrated manner, and develop local, culturally relevant, and context-specific solutions to address both undernutrition and the dual burden of malnutrition. Creative and innovative approaches using technology-based interventions may enhance access to services, promote caregiver involvement, and improve adherence to nutritional recommendations, especially in rural areas or locations without a lot of resources. Establishing comprehensive monitoring and evaluation systems that support the development of evidence-based changes to enhance programs, facilitate continuous improvement, and provide the potential for scaling up effective interventions should be the foundation of any program or policy (67).

## Conclusion

Various childhood nutrition interventions have been shown to substantially improve children’s growth, cognitive ability, and overall fitness, especially when the programs target vulnerable populations through multiple levels of action. Studies have shown that multisector, culturally appropriate, and equitable strategies to address the immediate nutritional needs of children and the long-term social and economic conditions that contribute to insufficient nutrition lead to the most significant reductions in health inequities. Facilities offering a range of services that target children and families, including policies, community, health care, and household-level programs, have demonstrated measurable improvements in anthropometrics, micronutrient levels, and cognitive function. While there has been success in improving the overall health status of children, there remains a lack of access to resources necessary to support vulnerable populations, particularly those living in rural communities without adequate resources, or in urban low-resource settings, which create and perpetuate intergenerational cycles of poor nutrition due to a combination of economic hardship, hunger, inadequate access to health care, and lack of caregiver education. Strategies that are coordinated, sustainable, and culturally sensitive are necessary in order to be successful. Policymakers, the NHS, and NGOs must work together to develop and implement interventions that are evidence-based, scalable, and use innovative technologies such as mobile health applications, digital nutrition education, and data-driven monitoring through a participatory process that engages the community and empowers caregivers. Using these multi-level and multi-sectoral approaches will allow societies to significantly reduce childhood nutrition disparities; provide equal access to basic nutrition; and support all children to grow optimally, develop their minds, and achieve lifelong health, while breaking the cycle of malnutrition for generations.
